# An Atomistic Model Describing the Structure and Morphology of Cu-Doped C-S-H Hardening Accelerator Nanoparticles

**DOI:** 10.3390/nano12030342

**Published:** 2022-01-21

**Authors:** Gregorio Dal Sasso, Maria Chiara Dalconi, Giorgio Ferrari, Jan Skov Pedersen, Sergio Tamburini, Federica Bertolotti, Antonietta Guagliardi, Marco Bruno, Luca Valentini, Gilberto Artioli

**Affiliations:** 1 Institute of Geosciences and Earth Resources, National Research Council of Italy, Via G. Gradenigo 6, 35131 Padova, Italy; 2 Department of Geosciences and CIRCe Centre, University of Padova, Via G. Gradenigo 6, 35131 Padova, Italy; mariachiara.dalconi@unipd.it (M.C.D.); luca.valentini@unipd.it (L.V.); gilberto.artioli@unipd.it (G.A.); 3 MAPEI Spa, R&D Laboratory, 20158 Milano, Italy; g.ferrari@mapei.it; 4 Department of Chemistry and Interdisciplinary Nanoscience Center (iNANO), Aarhus University, Gustav Wieds Vej 14, 8000 Aarhus, Denmark; jsp@chem.au.dk; 5 Institute of Condensed Matter Chemistry and Technologies for Energy, National Research Council of Italy, Corso Stati Uniti 4, 35127 Padova, Italy; sergio.tamburini@cnr.it; 6 Department of Science and High Technology and To.Sca.Lab, University of Insubria, Via Valleggio 11, 22100 Como, Italy; federica.bertolotti@uninsubria.it; 7 Institute of Crystallography and To.Sca.Lab, National Research Council of Italy, Via Valleggio 11, 22100 Como, Italy; antonella.guagliardi@ic.cnr.it; 8 Department of Earth Sciences, University of Turin, Via Valperga Caluso 35, 10125 Torino, Italy; marco.bruno@unito.it

**Keywords:** calcium silicate hydrate, cement hardening accelerator, Debye scattering equation, small-angle X-ray scattering, wide-angle X-ray total scattering, ^29^Si MAS-NMR, EXAFS, nanostructure

## Abstract

Calcium silicate hydrate (C-S-H) is the main binding phase in Portland cement. The addition of C-S-H nanoparticles as nucleation seeds has successfully been used to accelerate the hydration process and the precipitation of binding phases either in conventional Portland cement or in alternative binders. Indeed, the modulation of the hydration kinetics during the early-stage dissolution-precipitation reactions, by acting on the nucleation and growth of binding phases, improves the early strength development. The fine-tuning of concrete properties in terms of compressive strength and durability by designed structural modifications can be achieved through the detailed description of the reaction products at the atomic scale. The nano-sized, chemically complex and structurally disordered nature of these phases hamper their thorough structural characterization. To this aim, we implement a novel multi-scale approach by combining forefront small-angle X-ray scattering (SAXS) and synchrotron wide-angle X-ray total scattering (WAXTS) analyses for the characterization of Cu-doped C-S-H nanoparticles dispersed in a colloidal suspension, used as hardening accelerator. SAXS and WAXTS data were analyzed under a unified modeling approach by developing suitable atomistic models for C-S-H nanoparticles to be used to simulate the experimental X-ray scattering pattern through the Debye scattering equation. The optimization of atomistic models against the experimental pattern, together with complementary information on the structural local order from ^29^Si solid-state nuclear magnetic resonance and X-ray absorption spectroscopy, provided a comprehensive description of the structure, size and morphology of C-S-H nanoparticles from the atomic to the nanometer scale. C-S-H nanoparticles were modeled as an assembly of layers composed of 7-fold coordinated Ca atoms and decorated by silicate dimers and chains. The structural layers are a few tens of nanometers in length and width, with a crystal structure resembling that of a defective tobermorite, but lacking any ordering between stacking layers.

## 1. Introduction

Cementitious binders based on ordinary Portland cement (OPC) are complex, multiphase and nanostructured systems resulting from the reaction of a mixture of finely ground calcium silicates, aluminates and alumino-ferrites with water. The final product is a hardened paste constituted by several hydration products, the most abundant of which, and responsible for the chemical and mechanical properties of the binder, is a poorly crystalline calcium-silicate-hydrate commonly known as C-S-H [1]. The complexity of this system arises from the hydration of multiple phases, differing in chemical and mineralogical composition. Thus, the hydration process is regulated by a high number of parameters that influence the dissolution/precipitation kinetics of several phases in the aqueous environment [1]. Moreover, the particle size, the modulation of water-to-cement ratio and the use of chemical admixtures to modify the particle dispersion and the kinetics of the reaction during hydration directly influence the workability of the binder as well as the strength development and the microstructure of the hardened material [1,2,3,4]. The system complexity further increases when OPC is partially replaced by supplementary cementitious materials (SCMs), both as naturally occurring materials or as industrial by-products that take part in the pozzolanic reactions, or by formulating alternative OPC-free binders as the alkali-activated materials (AAMs) [5,6,7]. Indeed, in blended cements and alternative binders, the chemical-mineralogical and microstructural features of nanocrystalline/amorphous hydration products (namely C-A-S-H, C-N-A-S-H, N-A-S-H, …), which also incorporate Al and alkali metals as major components, depart from that of the C-S-H found in traditional cements [5,8]. Accessing in detail the compositional and structural features of these phases at the atomic and nanometer scale is crucial for deeply understanding the role of key parameters in determining the binders’ functional properties [9,10]. Thus, manipulating the structure and hydration kinetics by acting at the atomic-/nano-scale will enable the designing of binders with desired rheological and mechanical properties and durability at the macro-scale [1].

In this view, particularly meaningful is the development of cement hardening accelerators, a vast class of materials that chemically and/or physically accelerate the hydration kinetics of cementitious binders, promoting the dissolution of calcium silicates and modulating the nucleation and growth of C-S-H [11,12]. The increasing interest in speeding up the setting and strength development of cement pastes reflects the need for reducing the construction time and formwork removal in specific applications, as well as enabling the fast compressive strength development at low environmental temperatures. Particularly attractive for the long-term durability of the hardened binder is the use of seeding-additives, constituted by a dispersion of nanoparticles, intrinsically characterized by a high specific surface area, which acts as nucleation sites for C-S-H precipitation [12]. From a wider perspective, nucleation seeding may offer great opportunities to overcome the delaying effects in the early strength development occurring in blended cements due to the addition of SCMs or in alternative binders, because of the low reactivity of precursors, with respect to conventional OPC, enabling their effective deployment in large-scale applications [13,14]. Among seeding-additives, the use of C-S-H nanoparticles as nucleation seeds is very promising because of their high compatibility with cement materials, representing the ideal substrate to favor the precipitation of calcium silicate hydrates, with the beneficial effects of reducing the capillary porosity and improving the long-term strength and durability of the hardened paste [3,11,13,15,16,17].

The sample selected for this study is a cement hardening accelerator constituted by a colloidal suspension of Cu-doped C-S-H nanoparticles stabilized with polycarboxylate polymers (PCE). The feasibility of transition metals (Cu in this case) doping of C-S-H nano-seeds is a promising strategy to further develop seeding additives by favoring the functionalization of the surface of the nanoparticles with a variety of organic molecules, aiming at enhancing the mechanical properties in the hardened paste. To this aim, providing a detailed structural characterization of this material is crucial to achieve strict control on the nanostructure manipulation of cement pastes and to eventually modulate the desired mechanical properties.

The detailed description of cement materials is a challenging task because of their nano-sized, disordered, chemically complex and multiphase nature. Many improvements in the atomic-level description of poorly crystalline phases have been recently achieved through advanced analytical techniques coupled with thermodynamic modeling and molecular dynamics simulations; however, a comprehensive characterization is still a matter of debate [9,10,18,19,20,21,22,23,24]. On the one hand, detailed insights have been retrieved by using transmission electron microscopy and methods probing the chemical environment of atomic species and the short-range structural order, such as Fourier transform infrared (FTIR) and Raman spectroscopies, nuclear magnetic resonance (NMR), X-ray absorption and pair distribution function analysis of X-ray scattering data [9,19,21,23,25,26,27,28]. On the other hand, the lack of long-range structural order prevents taking full advantage of conventional X-ray diffraction methods and refinement algorithms for the structural characterization of these nano-phases [9,10,29,30]. Indeed, the diffraction pattern of nanocrystalline phases shows only few and broad Bragg peaks and a significant amount of diffuse scattering between and below the peaks, produced by the finite size and defectiveness of the crystallites [9,29]. Even though information on the average structure and unit cell parameters has been retrieved from X-ray powder diffraction data using Rietveld-based methods [29,31], the main limitation of such an approach, relying on long-range ordered structures, is the complete disregard of diffuse scattering, which contains information on the finite size, size distribution of crystallites and on the structural defectiveness [32]. Conversely, the wide-angle X-ray total scattering (WAXTS) approach is much more suitable because both the Bragg and diffuse scattering are equally treated and modeled, thus providing quantitative structural information at the atomic and nanometer length scale on nanostructured and disordered materials [32,33]. WAXTS data acquisition is usually performed at synchrotron beamlines, enabling the strict control on the extra-sample scattering and its successive subtraction from the WAXTS pattern, providing the sole sample contribution to the total X-ray scattering pattern. The data analysis and modeling can be either performed directly on the WAXTS trace in the reciprocal space using the Debye scattering equation (DSE) [34,35] or on the Fourier-transformed trace in the real space, through the aforementioned pair distribution function (PDF), analyzing the probability of finding pairs of atoms separated by a certain distance [36].

The increasing interest in applying total scattering methods to both conventional OPC and alternative binders, favored by the advances in both experimental setup and computing resources, reflects the relevance of unprecedented details that can be obtained on these materials at the atomic scale. Most studies have effectively applied PDF analysis, providing qualitative and quantitative information on the local atomic structure of C-S-H and other hydration products [9,10,24,37,38,39]. Alternatively, the DSE modeling of WAXTS data in the reciprocal space has been mostly disregarded.

Here, we implement a novel multi-scale approach to the characterization of cementitious materials that has been successfully applied to benchmark synthetic and biogenic/biomimetic nanomaterials [35,40,41,42], that is combining forefront small-angle X-ray scattering (SAXS) and synchrotron WAXTS methods. SAXS provides robust information on the nanoparticle size and morphology, whereas WAXTS accesses the atomic-scale features. The total scattering pattern in both the small- and wide-angle regions can be computed through the DSE from atomistic models, accounting for the structure and defectiveness as well as for the size and shape of nanoparticles. In this work, we aim to highlight the great potential of the DSE modeling approach to the detailed characterization of nanostructured cementitious materials, taking advantage of the recent improvement in computational efficiency [35,43]. With this approach, the probed local environment of atomic species was integrated into atomistic structural models of the entire nanocrystalline domains. Taking into account the available structural models for C-S-H (a brief overview is reported in Section 1.1), we developed novel atomistic models of Cu-doped C-S-H nanoparticles based on defective tobermorite, integrating local-structure information derived from ^29^Si magic-angle-spinning nuclear magnetic resonance (MAS-NMR) and Cu K-edge X-ray absorption spectroscopy. The modeling of both the SAXS and WAXTS data with a unified structural model through the DSE provided quantitative information on the structure, size and morphology of Cu-doped C-S-H nanoparticles, enabling a comprehensive description of the sample from the atomic to the nanometer scale.

### 1.1. A Brief Overview on Available C-S-H Structural Models

**Figure 1:**
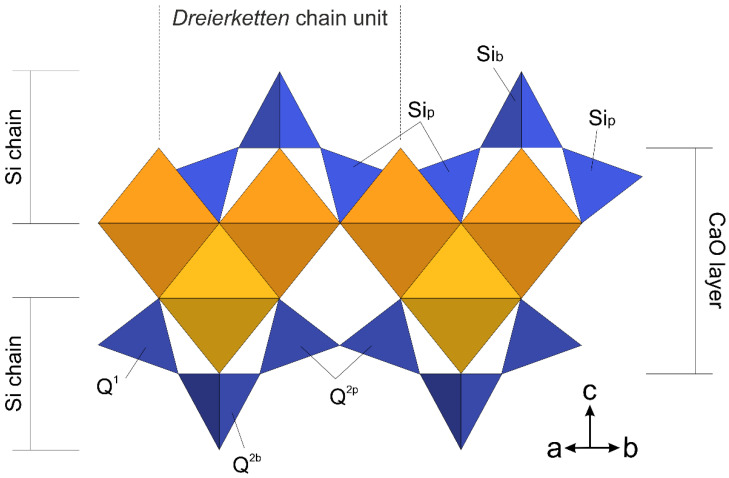
Sketch of the tobermorite-like structure of a layer of sevenfold-coordinated Ca^2+^ ions (orange) sandwiched by linear chains of silicate tetrahedra (blue) formed by repeating *dreierketten* chain units. Silicate bridging (Si_b_) and paired (Si_p_) tetrahedra are shown in the sketch. *Q*^n^ notation of silicate tetrahedra used in ^29^Si MAS-NMR analysis (see Section 3.4) is also reported.

Calcium silicate hydrate (C-S-H) has been extensively studied to understand its formation, structure and chemical-physical characteristics to be linked to the functional properties of the hardened cement paste [19,22,23,26,44,45,46]. It has a complex and variable chemical composition, manifested in a Ca/Si ratio ranging between 0.6 and 2.4, several types of ionic substitution and incorporation of foreign elements [22,45]. The nano-sized and structurally-disordered nature of C-S-H hindered a comprehensive description of its structure and a straightforward coupling between theoretical models and experimental data. These issues, together with the use of different methods encompassing molecular dynamic simulations, thermodynamic calculations, chemical and crystallographic characterization [19,20,23,47,48], led to the development of several structural models, mostly relying on the crystalline-phase counterparts [22,26]. X-ray diffraction patterns show few and broad peaks pointing towards a nanocrystalline disordered system rather than an amorphous one; nevertheless, classical Rietveld methods are unable to satisfactorily model the experimental data [19]. Therefore, alternative methods have been used to probe the local short-range order, such as FTIR, Raman and X-ray absorption spectroscopies and nuclear magnetic resonance. The general consensus points toward a structural model derived from that of tobermorite, a mineral phase with a chemical composition close to Ca_5_Si_6_O_17_·5H_2_O. The tobermorite structure consists of parallel stacking layers, each one constituted by a layer of sevenfold-coordinated Ca^2+^ ions decorated by linear silicate chains on both sides (Figure 1).

Silicate chains are formed by repeating *dreierketten* chain units made of two silicate tetrahedra (named paired tetrahedra and labeled as Si_p_), linked together and sharing O-O edges with the Ca polyhedra, and one silicate tetrahedron (named bridging tetrahedron and labeled as Si_b_) connecting two dimers of paired silicate tetrahedra and sharing an O atom with a Ca polyhedron [49]. The tobermorite supergroup is formed by a number of mineral phases characterized by different hydration states, classified on the basis of the layer-to-layer distance of 14, 11 and 9 Å. Among them, the crystal structure of several polytypes have been resolved [49,50,51,52,53]; in particular, the tobermorite 11 Å [49] and the tobermorite 14 Å (or plombierite) [51] better reflect the crystalline counterpart of nanocrystalline and defective C-S-H produced by the hydration of Portland cement [29]. Indeed, C-S-H shows a variable layer-to-layer distance between 14 and 11 Å, depending on the Ca/Si ratio, with several ions and water molecules bound to the structure in the interlayer space [54]. Recent studies favor a continuous structural evolution between C-S-H types, spanning the entire Ca/Si range, based on a tobermorite structural model [19,21,23,24,31,54,55], rather than a two-phase model based on the occurrence of tobermorite and jennite to describe C-S-H at low (<c.a. 1.3) and high (>c.a. 1.3) Ca/Si ratios, respectively [26,44]. Combining the measured Ca/Si ratio in C-S-H systems (well above the 0.83 of crystalline tobermorite) with the tobermorite-based structural model is still a matter of debate. To increase the Ca/Si ratio with respect to that of the tobermorite structure, besides the progressive elimination of bridging silicate tetrahedra, two models have been suggested: one is a binary nanocomposite system of C-S-H and amorphous/nanocrystalline Ca(OH)_2_ possibly located in the interlayer region [24], whereas the other suggests the substitution of protons with Ca^2+^ atoms in the interlayer, and eventually occupying the vacancy at Si_b_ sites bridging silicate paired tetrahedra [23]. Turbostratic stacking of layers has been introduced to account for the structural disorder among stacking layers at the nanoscale [29,54,56].

## 2. Materials and Methods

### 2.1. Synthesis of Me-S-H/PCE Nanocomposite—Mapefast Ultra

The Cu-doped C-S-H hardening accelerator (Me-S-H/PCE) is a colloidal suspension of copper-calcium (metal—Me) silicate (S) hydrate (H) nanoparticles stabilized with polycarboxylate polymers (PCE), commercially known as Mapefast Ultra (MAPEI S.p.A.). The Me-S-H/PCE nanocomposite is prepared by the double precipitation method by combining an aqueous solution of Cu(NO_3_)_2_ and Ca(NO_3_)_2_ with a Cu/Ca molar ratio of 0.1 (solution A), with an aqueous solution of Na_2_SiO_3_ (solution B), in the presence of MPEG-PCE superplasticizer. The MPEG-PCE superplasticizer was synthesized by free radical aqueous polymerization of a terpolymer of methoxy-polyethyleneglycol-methacrylate (molecular weight of about 5000 Da), acrylic acid and methacrylic acid. The final polymer had a molecular weight of 100,000 Da, as determined by gel filtration chromatography (GFC). At first, 1173 g of a MPEG-PCE aqueous solution (solids content 7.5 weight percent) were placed in a 2 L glass-bottom rounded reactor equipped with mechanical stirrer. Then, 398 g of solution A, containing 1 mol of Ca(NO_3_)_2_ and 0.1 mol of Cu(NO_3_)_2_, and 550 g of solution B, containing 1.1 mol of Na_2_SiO_3_, were simultaneously fed to the reactor while stirring at 300 rpm at the temperature of 20 °C. The addition of solutions A and B was completed in 1 h; then, the suspension was stirred for one additional hour and then discharged. The final suspension, of light blue color, had a total solid content of 23 wt.% and a viscosity of 0.12 N·s·m^−2^, measured at 20 °C. Previous sample characterization certifies the effective synthesis of nano-sized particles of a few tens of nanometers in diameter [3].

### 2.2. Small-Angle X-ray Scattering (SAXS)

The SAXS measurement was performed on the in-house SAXS instrument at Aarhus University [57], equipped with a rotating Cu anode source, side-by-side Montel multilayer mirrors to monochromatize and focus the beam, and a Vantec 500 (Bruker AXS, East Cheryl Parkway, Madison, WI, USA) detector upgrade. The collimation consists of two pinholes, where the one close to the sample is a scatterless pinhole with edges of Ge crystal [58,59]. The Me-S-H/PCE suspension was mounted in a glass capillary and measured. The empty glass capillary was also measured and subtracted as background. The data treatment was done using the in-house developed SUPERSAXS program package (C.L.P. Oliveira and J.S. Pedersen, unpublished). The SAXS experimental pattern was acquired in the 0.01–0.37 Å^−1^ *Q* range (scattering vector amplitude *Q* = 4πsinθ/λ, where λ is the X-ray wavelength and 2θ is the scattering angle). Conventional SAXS analysis was performed using an analytical form factor. The SAXS data were modeled as polydisperse flat cylindrical discs [60] using a Schulz distribution for the number size distribution [61]. The same polydispersity distribution was assumed in both radius and thickness; however, with a 10 times smaller polydispersity (*σT*/*T*) of the thickness (*T*). The SAXS data had clear signs of particle agglomeration as a power-law with an exponent of approximately −4 observed at low *Q*. A scale factor for this contribution and a constant background were included as fit parameters in addition to an overall scale factor and the average radius *R*, the average thickness *T* and the relative polydispersity σ_rel_. The parameters were optimized using a weighted least-squares method [62]. 

### 2.3. Synchrotron Wide Angle X-ray Total Scattering (WAXTS)

The Me-S-H/PCE suspension was loaded into a 0.5 mm diameter glass capillary and measured at the X04SA-MS beamline of the Swiss Light Source (Paul Scherrer Institut, Villigen, CH, Switzerland) [63]. WAXTS data were collected in transmission mode in the 2θ range 2–120° using a single-photon counting silicon microstrip MYTHEN II detector. The beam energy was set at 22 KeV and operational wavelength λ = 0.56456 Å, determined by measuring a silicon powder standard sample (NIST 640c). The Me-S-H/PCE was centrifuged at high speed to isolate the dispersing solution, which was loaded in a glass capillary and measured separately. Separate air and empty capillary scattering measurements were also acquired. The transmission coefficient of the sample was experimentally determined by measuring the direct and transmitted beams, whereas that of the glass capillary was calculated from the certified composition and wall thickness of the capillary. Raw data were corrected for systematic errors and absorption effects; the extra-sample contributions to the scattering pattern, i.e., the scattering from the capillary and the sample environment, were subtracted. The reduced WAXTS data containing only the sample scattering pattern was analyzed by the total scattering approach based on the Debye scattering equation (DSE), which is described further in the next section.

### 2.4. The Debye Scattering Equation (DSE) Method

The efficient implementation of the Debye scattering equation [34] within the DebUsSy Suite of programs [35] enables the computation of the total X-ray scattering pattern in the reciprocal space of randomly oriented and non-interacting nanoparticles from the distribution of interatomic distances within atomistic models. Unlike conventional Rietveld methods, the DSE modeling approach does not require any assumption on structural periodicity, relying on atomistic models that account for the finite size and structural defectiveness of nanocrystalline materials. The simulated scattering pattern is calculated as:

(1) $$I\left(Q\right)=\overset{N}{\underset{j=1}{\sum }}f_{j}{\left(Q\right)}^{2}o_{j}{}^{2}+2\overset{N}{\underset{j>i=1}{\sum }}f_{j}\left(Q\right)f_{i}\left(Q\right)T_{j}\left(Q\right)T_{i}\left(Q\right)o_{j}o_{i}\frac{\sin\;\left(Qd_{ij}\right)}{\left(Qd_{ij}\right)}$$

where *f_j_*(*Q*) is the atomic form factor of atom *j*, *d_ij_* is the interatomic distance between atom *i* and *j* and *N* is the number of atoms in the nanoparticle. *T_i_*(*Q*) and *o_i_* parameters refer to the atomic thermal vibration and site occupancy, respectively. The first summation accounts for the contribution of the zero distances of each atom from itself, whereas the second summation accounts for the non-zero distances between pairs of distinct atoms. The DSE modeling is carried through a bottom-up approach. The unit cell derived from the crystal structure of tobermorite is used as the building block to generate a population of atomistic models of nanocrystals of increasing size and desired shape. For each nanocrystal model, sampled interatomic distances are calculated and encoded in a database used to simulate the diffraction pattern through the Debye equation. A sampling algorithm of the true distances reduces by order of magnitude the number of terms in the Debye equation without losing accuracy in the calculated pattern, making the analysis affordable in terms of computational time [43]. The calculated scattering pattern is obtained as the weighted sum of the patterns from each atomistic model according to their number fraction of bivariate lognormal size distribution. The calculated pattern was then compared to the experimental one, and the difference between them was minimized using a simplex algorithm by refining a number of adjustable structural and microstructural parameters in the model [35]. The number-based average size and size dispersion along two independent growth directions are optimized according to a bivariate lognormal function (D, equivalent diameter of the area in the a-b plane, and length along the c axis). The isotropic atomic thermal displacement (Debye-Waller *B* factor) and the site occupancy factors (s.o.f. ***o_i_***) are also refined. The goodness of fit (GoF, the square root of reduced χ^2^) is the statistical descriptor used to evaluate the agreement between calculated and experimental patterns. The construction details of the atomistic models are reported in Section 3 of this article. The DSE modeling of scattering data is usually carried out in the wide-angle region (where atomic-scale details are investigated), but it can also be successfully performed in the small-angle region (where nanometer-scale details are explored) [40,42,64].

### 2.5. ^29^Si Magic-Angle-Spinning Nuclear Magnetic Resonance (NMR)

^29^Si spectra were collected on a Bruker AVANCE III 300 spectrometer, operating with a magnetic field of 7.0 T corresponding to a ^29^Si Larmor frequency of 59.623 MHz and equipped for solid-state analysis in 4 mm diameter zirconia rotors with Kel-F caps. The magic angle was accurately adjusted prior to data acquisition using KBr. ^29^Si chemical shifts were externally referenced to solid tetrakis(trimetylsilyl)silane at –9.0 ppm (in relation to TMS). The quantitative ^29^Si single-pulse experiments were collected at a spinning frequency of 6 kHz, a recycling delay of 120 s and 5000 transients. ^29^Si spectra analysis was performed through spectral deconvolution using combined Gaussian-Lorentzian peak functions with Dmfit software (Software 2021, Oleans, France) [65].

### 2.6. Cu K-Edge X-ray Absorption Spectroscopy (XAS)

XAS spectra at the Cu K-edge were collected at the XAFS beamline of the Elettra Synchrotron light source (Elettra-Sincrotrone Trieste S.C.p.A, Trieste, Italy) [66]. The Me-S-H/PCE sample was measured as a colloidal suspension enclosed between two kapton foils and as a dried powder pressed into pellets. Samples were measured in transmission mode at liquid-nitrogen temperature using energy scanning with a variable step: large step (5 eV) in the first 200 eV of the spectrum, smaller step (0.2 eV) in the XANES (X-ray absorption near edge structure) region and a wave vector k-constant step of 0.03 Å^−1^ in the EXAFS (extended X-ray absorption fine structure) region. Energy calibration was attained by simultaneously collecting a reference spectrum of a Cu metal foil placed in a second experimental chamber after the sample. EXAFS spectra were processed and analyzed using the Athena and Artemis programs suite (https://bruceravel.github.io/demeter; accessed on 15 January 2022) [67].

## 3. Results and Discussion

### 3.1. Defining a Suitable Atomistic Model for Me-S-H Nanoparticles

The Me-S-H/PCE WAXTS pattern showed few and broad peaks compatible with those produced by a nanocrystalline tobermorite structure (Figure S1). According to previously developed structural models for C-S-H [19,21,23,24,31,54,55], both the structures of tobermorite 11 Å from Merlino et al. (2001) [49] and tobermorite 14 Å from Bonaccorsi et al. (2005) [51] were selected to build atomistic models to be used to analyze the Me-S-H/PCE sample through the DSE method.

Preliminary DSE modeling using either the tobermorite 11 Å or 14 Å showed an unsatisfactory fit of the experimental data after adjusting the size and size dispersion of nanoparticles (as indicated by the high GoF caused by the mismatch between the experimental and calculated patterns, visually highlighted by the residual plot—Supplementary Materials, Figure S2a,b). In detail, the calculated patterns showed a higher degree of order than in the experimental one, with a number of additional peaks in the angular positions compatible with the contribution of 00l reflections observable in the diffraction pattern of crystalline tobermorite. Increasing the peak broadening of the calculated WAXTS pattern by reducing the average diameter and/or modifying the size dispersion of the nanoparticles model was not enough to fit the experimental pattern, suggesting that the structural models based on the unit cell content of tobermorite 11 Å and tobermorite 14 Å are not fully suitable to describe the Me-S-H/PCE WAXTS pattern.

Thus, a novel atomistic model was built, disregarding any structural order between the tobermorite stacking layers, supported by the fact that: (i) in the low-angle region of the WAXTS pattern (Figure S1), in the 0.45–0.50 Å^−1^ *Q* range, the basal (002) reflection characteristic of the layer-to-layer distance periodicity of tobermorite structures was completely missing; (ii) SAXS analysis (*vide infra*) provides a particles thickness of ~1.17 nm, compatible with the thickness of a single Ca layer decorated with silicate chains constituting half (along the layers stacking direction) of the tobermorite unit cell. The atomistic model was built (hereafter, single-layer model) using only half of the unit cell content of the tobermorite 11 Å [49], where a single Ca layer is sandwiched between silicate chains and water molecules; from this new unit cell, a disc-shaped population of atomistic models of (single-layer) nanoparticles was built at increasing size in the *a-b* plane.

The preliminary DSE simulation from the single-layer model showed a relevant GoF improvement and a much better agreement with the experimental pattern (Supplementary Materials, Figure S2c), making this the model of choice to be further developed in this study. Indeed, the single-layer model accounts for the thickness of nanoparticles retrieved from SAXS analysis and provides a simulated WAXTS pattern with suppressed (00l) contributions, in agreement with the experimental data.

The *a* and *b* cell parameters of this new unit cell were optimized against the experimental WAXTS pattern, obtaining the values *a* = 6.7118(3) Å and *b* = 7.3397(2) Å, with a slight contraction with respect to the crystalline tobermorite 11 Å. The atomic positions of silicate tetrahedra and water molecules (as occurring in the unit cell of the tobermorite 11 Å [49]) were optimized through density functional theory calculations (see the Supplementary Materials for a detailed description of the optimization method and for the atomic coordinates in the unit cell). Based on the resulting unit cell, a population of disc-shaped atomistic models of (single-layer) nanocrystals was generated according to a cylindrical growth model by increasing the diameter of the cylindrical base (laying in the *a-b* plane, with a size discretization of 7.25 Å) and constant height (equal to the *c* unit cell parameter), and used in the following DSE analysis of both SAXS and WAXTS data.

### 3.2. Me-S-H Nanoparticles Size and Morphology by SAXS Analysis

#### 3.2.1. Conventional SAXS Modeling

**Figure 2:**
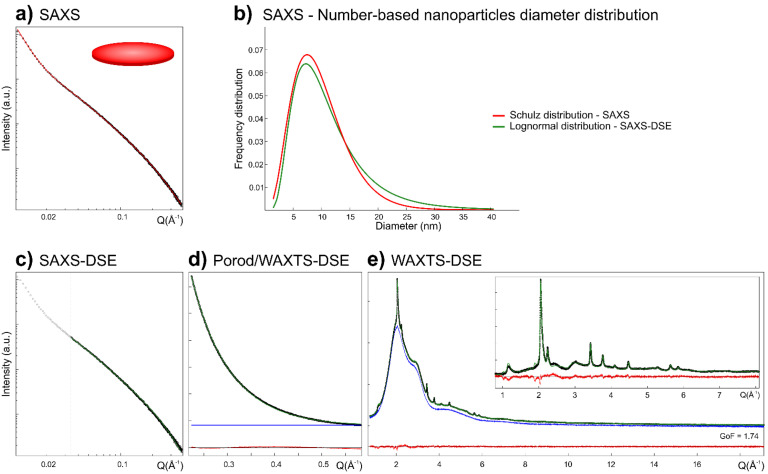
(**a**) SAXS data (black dots, log-log scale) of Me-S-H nanoparticles and best fit (solid red line) from analytical form factor using the disc-shaped model; (**b**) comparison between the number-based diameter size distributions obtained by conventional SAXS analysis using a Schulz distribution (red line) and by the DSE modeling approach using a lognormal distribution (green line); (**c**) SAXS data (black dots, log-log scale) and (**d**,**e**) synchrotron WAXTS data (black dots) of Me-S-H nanoparticles. In (**c**–**e**), the solid green line is the best fit obtained through the DSE using the single-layer atomistic model. The WAXTS pattern of the dispersing solution (blue trace) was added as a model component and scaled to the experimental data. The red line is the residual between experimental and calculated patterns. The inset reports the WAXTS pattern and the DSE fit in a limited *Q* range by subtracting the scattering contribution of the solution.

The size and morphology of Me-S-H nanoparticles were determined through SAXS analysis using the classical modeling approach. The scattered intensity was calculated from an analytical form factor, describing the size and shape of nanoparticles, and from a structure factor, accounting for the particle-particle interaction due to concentration or aggregation effects. Given the higher average electron density of mineral nanoparticles, their contribution to the SAXS pattern was assumed to be the dominant one, making that from PCE polymers negligible. The SAXS data were acquired and analyzed in the 0.01–0.37 Å^−1^ *Q* range. Model parameters were optimized by least-square methods against the experimental data. The best fit (χ^2^ = 7.68, Figure 2a) of the experimental SAXS data was obtained using a flat cylindrical disc model to describe the morphology of Me-S-H nanoparticles. The optimized model relies on a population of ultra-thin disc-shaped nanoparticles with a high polydispersity in diameter, with sizes up to about 44 nm. In detail, Me-S-H nanoparticles had a number-average diameter *D* = 9.78 nm, with a relative size dispersion σ*_D_*/*D* = 0.49, whereas the number-average thickness *T* = 1.17 nm showed a much narrower size dispersion σ*_T_*/*T* = 0.05 (which has been kept at a 10 times smaller value than for the diameter), using a Schulz distribution (Table S1). In the low *Q* range of SAXS data (below 0.025 Å^−1^), the slope change of SAXS data in a log-log representation indicated the occurrence of inter-particle interactions; thus, a power-law with an exponent of c.a. −4 was introduced to account for particle aggregation effects. The thin platy morphology of Me-S-H nanoparticles is well compatible with the single layers of tobermorite structure used to describe the C-S-H systems [21].

#### 3.2.2. SAXS Modeling Based on the DSE

The computation of the Debye scattering equation in the low *Q* range to model the SAXS data is an emerging approach to the morphological characterization of nanomaterials, complementary to the DSE modeling at higher *Q* of WAXTS data [40,41,42,64,68]. Even though the SAXS signal is not sensitive to the atomic scale features, the DSE enables the computation of the SAXS pattern from atomistic models, providing information on the size and morphology of nanoparticles. The DSE modeling does not account for particle-particle interactions so that it can be reasonably applied when aggregation and concentration effects are negligible or by omitting the part of the data that are influenced by these effects. Despite the high computational costs, the DSE modeling of SAXS data has the great advantage that the same size discretization and distribution law of atomistic models can be used to compute both the SAXS and WAXTS patterns enabling the prompt transfer of information on optimized parameters from the small- to wide-angle region, i.e., once provided the size and size dispersion of nanoparticles, other structural parameters can be safely refined in the DSE analysis of WAXTS data. Thus, the combined analysis of SAXS and WAXTS data under a unified modeling approach, through the DSE, enables a detailed description of nanoparticles from the atomic- to the nanoscale.

Since the DSE analysis relies on the assumption of non-interfering particles, the DSE modeling of the Me-S-H SAXS pattern was carried out in the 0.025–0.37 Å^−1^ *Q* range, disregarding the SAXS signal at lower *Q* (0.01–0.025 Å^−1^) dominated by concentration/aggregation effects. Data truncation, although widely used, may not be the most accurate way to deal with undesired concentration effects as the concentration contribution occurs in the whole *Q* range, even though minimal at medium- and high-*Q* values, and the recovery of structural information may be hampered by an excessively limited *Q* range [69]. Nevertheless, in this case, it can be considered an acceptable approximation for the DSE modeling, since results can be compared (and supported) with those obtained from the conventional SAXS modeling approach, for which the scattering contribution of aggregates has been accounted for in the entire *Q* range by including a power law in the model.

The DSE pattern was calculated using the single-layer atomistic model previously defined, for which the average diameter size and dispersion, according to a lognormal size distribution law, were refined against the experimental data (Results in Supplementary Table S1). The best fit (χ^2^ = 1.27, GoF = 1.13, Figure 2c) was obtained through a number-based size distribution of nanoparticles with average diameter *D* = 11.24 nm and a relative size dispersion σ*_D_*/*D* = 0.59 (the corresponding mass-based distribution had an average diameter *D* = 20.20 nm and a relative size dispersion σ_D_/*D* = 0.49). The nanoparticles thickness was *T* = 1.13 nm. The specific surface area SSA = 1013 m^2^g^−1^ was calculated from the size of each nanoparticle weighted by its number-based distribution fraction. When comparing the number-based diameter size distributions obtained from the SAXS analysis based on the conventional approach and on the DSE-based one (Figure 2b), results showed negligible discrepancies in terms of average size and size dispersion, possibly accounting for the different distribution law, size discretization and thickness dispersion between the two approaches. The non-refinable and ideally monodisperse thickness of nanoparticles in the DSE modeling approach, resulting from the thickness of the tobermorite-like layer, was in reasonable agreement with the nanoparticle thickness obtained by the conventional modeling approach, for which structural constraints in the construction of the disc-shaped particle was missing. Notably, even though SAXS is blind to the atomic scale, data analysis through the DSE, integrating the structural information from WAXTS data (i.e., monolayer model derived from the tobermorite structure), can ease the coupling between the morphological details and the structural orientation of nanoparticles, thus identifying the growth direction of nanocrystals in the *a-b* plane of tobermorite layers. Further evidence of the model suitability to describe Me-S-H nanoparticles was provided by the DSE simulation in the Porod region at higher *Q* (0.22–0.58 Å^−1^), using the same model parameters (diameter size and distribution), which well fits the experimental data acquired in the low-angle region of the WAXTS pattern (Figure 2d). Model parameters on the average size and size distribution of nanoparticles obtained from the SAXS-DSE analysis have been promptly transferred to the WAXTS-DSE data modeling and used as a constraint during the analysis.

### 3.3. WAXTS-DSE Analysis of Me-S-H Nanoparticles

Synchrotron WAXTS data were collected in the 0.2–19.7 Å^−1^ *Q* range. The Me-S-H/PCE sample was measured as a colloidal suspension of nanoparticles; then, after centrifugation, the dispersing solution was separately measured. The WAXTS analysis based on the Debye equation enables the Bragg and diffuse scattering to be equally treated, enabling the simulation of the total scattering from the sample, accounting for the structure and defectiveness as well as for the size and shape of nanoparticles.

The Me-S-H/PCE WAXTS signal showed the typical diffraction pattern of nanocrystalline tobermorite (Figure S1) with few and broad Bragg peaks and a significant amount of diffuse scattering, most of which is compatible with the signal of the aqueous dispersing solution. The single-layer atomistic model (derived from tobermorite 11 Å structure and previously discussed) was used here to simulate the WAXTS pattern of Me-S-H nanoparticles through the DSE. The contribution of the dispersing solution was accounted for by adding its experimental WAXTS pattern (separately measured) as a model component and treated as a blank curve, suitably scaled to the Me-S-H WAXTS pattern, together with the simulated DSE pattern of the nanocrystalline phase.

A number of model parameters were adjusted and refined by minimizing the difference between the calculated and experimental patterns (Supplementary Tables S1 and S2). Since SAXS analysis provided reasonable information on the size and morphology of Me-S-H nanoparticles, the average (number-based) diameter size *D* and its relative dispersion σ*_D_*/*D* obtained from the SAXS-DSE analysis were kept fixed at the same values in the WAXTS-DSE analysis. Thus, by constraining the scattering features due to the size and shape of nanoparticles, details on structural defectiveness could be more safely refined. Site occupancy (0 < s.o.f. < 1) and Debye-Waller factors for each atomic species were separately refined; moreover, Si ions Si_b_ and Si_p_, as well as O atoms in the Ca layer, O in the silicate chains and the apical O in the Si_b_ tetrahedra were considered as distinct atomic species and treated separately. The four water molecules and OH^-^ ions were approximated to single O atoms, each of them treated separately. Refining the site occupancy and Debye-Waller factors of the atomic species accounts for the effects on the scattering pattern (averaged among all the atomic sites in the atomistic models) due to the occurrence of randomly distributed defectiveness (vacancies and/or spatial displacement) in the nanocrystals structure. The best fit (GoF = 1.74, Figure 2e) shows a very good agreement between the calculated and experimental data (refined site occupancy and Debye-Waller factors for each atomic species are reported in Table S2). The backbone of the Me-S-H structure is represented by the Ca-O layer characterized by full atomic site occupancy and low thermal displacement parameters. Conversely, silicate chains are much more fragmented due to the occurrence of randomly distributed vacancies (missing silicate tetrahedra), as shown by the partial occupancy of both the Si_p_ site (0.84) and the Si_b_ site (0.64), the latter with a higher thermal displacement parameter suggesting a much higher disorder in the position of the silicate bridging tetrahedra. The O atoms of the three water molecules at the surface of the nanoparticles show high thermal displacement parameters and partial occupancy (one of the O completely disappears); the correlating effects of the two parameters highlight the disordered distribution of water molecules, presumably occurring at the surface, as reported for C-S-H systems [54]. The excellent fit of both SAXS and WAXTS data shows the nanocrystalline nature of Me-S-H nanoparticles, as no evidence supports the occurrence of an amorphous component. Robust results highlight the suitability of the single-layer atomistic model developed so far, to describe the Me-S-H sample. Indeed, this model is compatible with C-S-H models suggested in the recent literature and based on disordered tobermorite structure [19,20,23,54,56]; nevertheless, two distinctive features are emerging: (i) an increasing number of studies [30,54,56] is suggesting that turbostratic disorder between stacking layers of the tobermorite structure accounts for the peak shape and broadening of the C-S-H diffraction pattern. In the case of the Me-S-H sample, the further increase of structural disorder with respect to the turbostratic model is evidenced by the lack of any stacking order between the single tobermorite-like layers, occurring as randomly-oriented nanocrystals. This can be primarily related to the synthesis procedure, combining the effects of mechanical stirring with the use of PCE molecules that feasibly decorate the surface of Me-S-H nanoparticles, preventing an ordered stacking of single layers as occurring in a typical tobermorite structure. This hypothesis is not contrasting the aggregation effects detected by SAXS analysis, as the measured suspension is not in ideal diluted conditions preventing the scattering contribution due to concentration-related particle-particle interactions. Nonetheless, despite the role of PCE in inducing the formation of a stable suspension and preventing flocculation, sufficiently small (to be stable in suspension) nanoparticle aggregates may form, albeit in a disordered configuration. The concept of interlayer space found in crystalline tobermorite or more structured C-S-H samples, accommodating a number of molecules and ionic species between stacking layers [23], is fading out, favoring alternative descriptions of particle-particle interactions controlled by the surface properties of individual nanoparticles. This model may be easily transferred to C-S-H systems characterized by structural disorder in the stacking layers, evaluating the role of single-layer nanoparticles at early stages of precipitation and their progressive spatial arrangement at longer times. (ii) The fragmentation of silicate chains is not only caused by the loss of bridging tetrahedra (here, 36% of them is missing), as largely attested, but also by a significant vacancy of silicate paired tetrahedra (here, at 16%), which has been scarcely considered by other models [20,70]. A detailed discussion on silicate tetrahedra connectivity is carried out in the next section through the analysis of the ^29^Si MAS-NMR spectrum acquired on the Me-S-H/PCE sample and by integrating the results into the single-layer atomistic model.

Lastly, the WAXTS-DSE analysis is further developed in Section 3.5 by exploring the feasibility of Cu incorporation into the structural model of Me-S-H nanoparticles by discussing the results obtained from Cu K-edge X-ray absorption spectroscopy.

### 3.4. Combining ^29^Si MAS-NMR and WAXTS-DSE Analyses to Describe the Silicate Tetrahedra Connectivity

**Figure 3:**
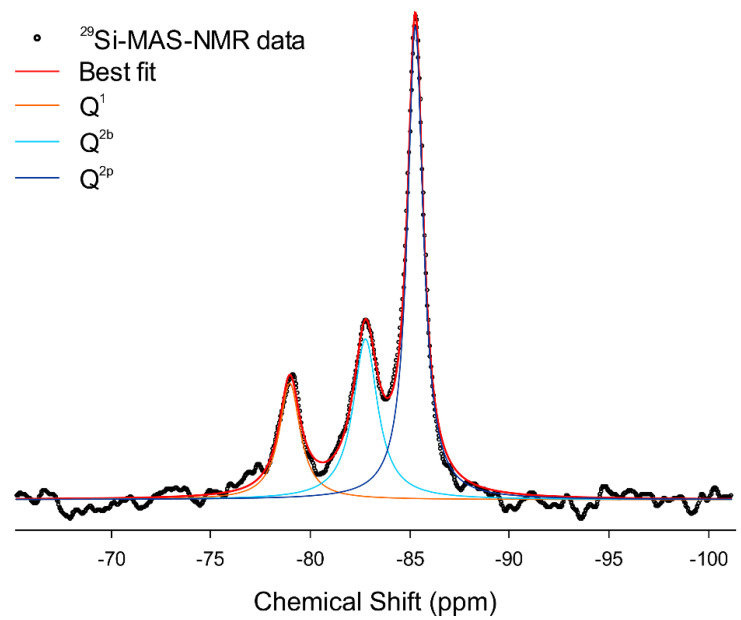
Solid-state ^29^Si MAS-NMR data (black dots) of Me-S-H sample and best fit (red line). Spectral deconvolution (R^2^ = 0.9936) was performed using three peaks at −78.96, −82.78 and −85.28 ppm attributed to Q^1^, Q^2b^ and Q^2p^ silicate tetrahedra, respectively.

Solid-state ^29^Si MAS-NMR spectroscopy is extensively used to probe the Si local chemical environment in cementitious materials, obtaining information on the silicate tetrahedra connectivity. Si sites in tetrahedral coordination are conventionally identified using the notation *Q*^n^ (not to be confused with the scattering vector amplitude *Q*, previously defined for the SAXS and WAXTS analyses), where *Q* is the Si atom bonded to *n* (with 0 ≤ *n* ≤ 4) other silicate tetrahedra (Figure 1). The typical chemical shift of ^29^Si in cementitious materials ranges from approximately −60 to −120 ppm, with a more negative chemical shift when increasing the connectivity [25]. The ^29^Si MAS-NMR spectrum acquired for Me-S-H/PCE (Figure 3) showed three distinct peaks at −78.96, −82.78 and −85.28 ppm, the first of which was attributed to the *Q*^1^ site (SiO_4_ bonded to one other silicate tetrahedra) and the other two to *Q*^2^ sites (SiO_4_ bonded to two other silicate tetrahedra).

As for Q^2^ sites, ^29^Si MAS-NMR enables the ^29^Si nuclei in bridging (indicated as *Q*^2b^) and paired (indicated as *Q*^2p^) silicate tetrahedra, occurring in the *dreierketten* chain unit, to be distinguished, as they resonate at −82.78 and −85.28 ppm, respectively. Evidence indicating a higher polymerization degree of the silicate chains is missing; as expected, *Q*^3^ characteristics of cross-linked sites occurring in mature C-S-H samples connecting tobermorite layers were not detected. Spectral deconvolution (R^2^ = 0.9936, Figure 3) was performed using three peaks at −78.96, −82.78 and −85.28 ppm, the integration of which provided the relative abundance of *Q*^1^, *Q*^2b^ and *Q*^2p^ in the sample, being 17.7%, 28.4% and 53.9%, respectively. These results can be used to calculate the mean silicate chain length (*MCL*), expressed in terms of the mean number of connected tetrahedra:

(2) $$MCL=2\left(1+\frac{P\left(Q^{2}\right)}{P\left(Q^{1}\right)}\right)$$

where *P*(*Q*^n^) is the population of *Q*^n^ species, providing an average indication of the polymerization degree of silicate chains [25]. The *MCL* obtained here was equal to 11.3 (number of connected tetrahedra, roughly corresponding to c.a. 2.6 nm), indicating the occurrence of shorter chains with respect to the nanoparticle diameters retrieved from SAXS and SAXS-DSE analyses.

Valuable information on tetrahedra connectivity is derived from solid-state NMR analysis, probing the local chemical environment of ^29^Si nuclei regardless of the structural order/disorder of the analyzed sample. Extending such information to defects distribution at the nanometer scale is not straightforward; indeed, few recent studies are addressing this issue by coupling atomistic model construction and results from solid-state NMR [70]. In this view, here we explored the feasibility of integrating the relative abundance of *Q*^n^ species from NMR analysis, averaged on the probed sample, in the single-layer atomistic model used in the SAXS/WAXTS-DSE analysis to achieve a more detailed description of the structural defectiveness in Me-S-H nanoparticles. In the WAXTS-DSE analysis previously discussed, the average effect of punctual (randomly distributed) silicate chain defectiveness was accounted for by refining the overall site occupancy factors of the Si_b_ and Si_p_ sites. Being both NMR and WAXTS signals averaged on the volume sample, the basic idea is to correlate the occupancy of Si sites to the measured *Q*^n^ species. To this aim, the relative abundance of *Q*^n^ species was simulated (indicated as *Q*^n’^) from the number of silicate tetrahedra occurring in each atomistic model of nanocrystal (weighted for its number-based size distribution fraction of the population of nanocrystals) and compared to the measured *Q*^n^ species. The site occupancy factors, derived from the WAXTS-DSE analysis, were used to assess the (average) vacancy at the Si_b_ and Si_p_ sites; then, depending on the vacancy type and extent, the average number of newly generated *Q*^1’^, *Q*^2b’^ and *Q*^2p’^ sites were calculated (see the details on *Q*^n’^ simulation in the Supplementary Materials).

Thus, the simulated *Q*^n’^ abundances, directly resulting from the WAXTS-DSE-derived Si_b_ and Si_p_ site occupancy factors were matched to the experimental *Q*^n^ from NMR analysis by optimizing the model parameter *A* (a numerical factor, ranging from 0 to 1, that modulates the number of newly generated *Q*^1^ sites by considering adjoining Si vacancies. See the details in the Supplementary Materials) through the least-squares method. The best agreement (residual sum of squares—RSS = 1.020) was reached for *A* = 0.39, providing *Q*^1’^ = 18.1%, Q^2b’^ = 27.5% and Q^2p’^ = 54.3%. Considering that, in the case of isolated vacancies, for each Si_b_ vacancy two *Q*^1^ sites are generated, this result means that, on average, about 0.8 *Q*^1^ sites are generated for each Si_b_ vacancy, thus implying the occurrence of neighboring vacancies. This indicates the occurrence of larger gaps in the silicate chains and a lower degree of fragmentation, a more energetically favored defect configuration with respect to highly fragmented chains [70]. Further discussing this approach, once defined the atomistic model, the agreement between calculated and experimental *Q*^n^ can be optimized by acting on three parameters (Si_p_Occ_, Si_b_Occ_ and *A*), of which two are directly derived from the WAXTS-DSE analysis. Considering that the partial occupancy of the silicate bridging site is coupled to a higher thermal parameter (meaning higher disorder, as discussed in Section 3.3), one can improve the agreement between the calculated and experimental *Q*^n^ by relaxing the Si_b_Occ_ parameter, while keeping fixed the more robust Si_p_Occ_ parameter. Thus, keeping the Si_p_Occ_ fixed at 0.84, a better agreement (RSS = 0.006) was obtained with minimal variation of the other two parameters, resulting in Si_b_Occ_ = 0.66 and *A* = 0.42. Even though the roughness of the Q^n’^ calculation proposed here, this exercise aimed at showing the great potential of integrating different techniques into a single and comprehensive modeling approach, which can be further improved in future studies. Indeed, integrating NMR results into the atomistic model can also provide some constraints to silicate chain defectiveness to safely refine the model parameters. In this view, we used the NMR results within an integrated approach with the SAXS/WAXTS-DSE analysis, aiming at further developing the single-layer atomistic model for Me-S-H nanoparticles by constraining the Si site occupancy and exploring the feasibility of Cu incorporation into the Me-S-H structure, taking into account the results from Cu K-edge X-ray absorption spectroscopy (see the following Section 3.5).

### 3.5. On the Effective Copper Incorporation into the Me-S-H Structure

**Figure 4:**
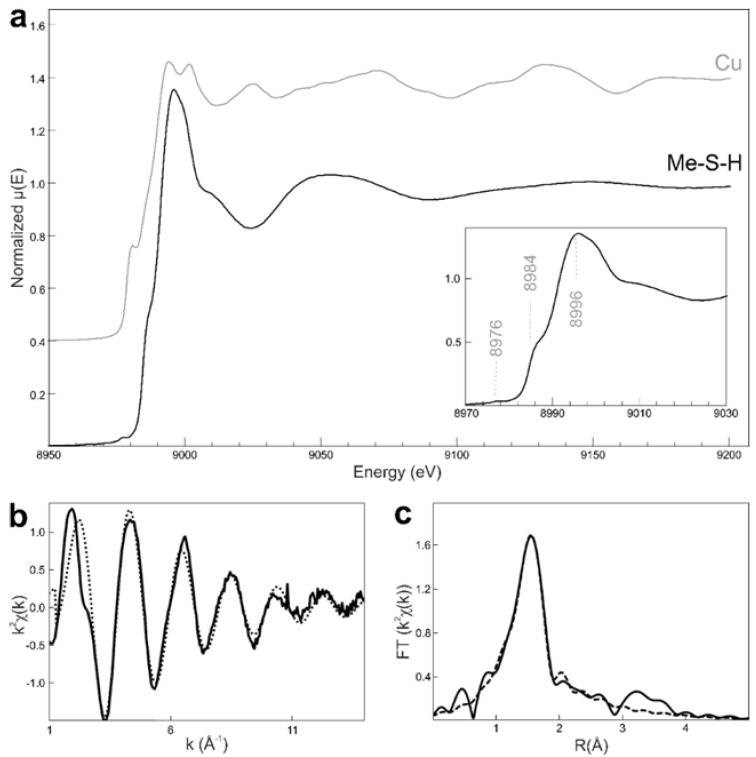
(**a**) Copper K-edge normalized spectrum of Me-S-H. A limited energy range is displayed to emphasize the XANES region. The normalized spectrum of Cu foil is reported for comparison. In the inset, a magnification of the Me-S-H spectrum to highlight the pre-edge and edge features (**b**) K^2^-weighted EXAFS signal at Cu k-edge and (**c**) magnitude of the corresponding Fourier transform (right). Solid lines are the measured signal; dotted lines are the fitted signal.

The Me-S-H sample, as well as the sample after high-speed centrifugation, has a light blue color, whereas the centrifuged dispersing solution is completely transparent. Despite this hint, suggesting that copper is bound to the solid fraction of the sample, evidence of its structural incorporation is missing. Thus, Cu K-edge X-ray absorption spectroscopy was performed, aiming at acquiring information on the chemical environment of Cu atoms in the sample. The XAS signals measured on the sample in the form of colloidal suspension and dried powder revealed to be identical (experimental traces are reported in Figure S3). This highlights the fact that the Cu local environment was not altered during drying; additionally, considering that the centrifuged dispersing solution was transparent, the presence of copper species in solution can be reasonably excluded. Three edge features were visible in the XANES region of the normalized XAS spectrum (Figure 4a): an intense white line at 8996 eV, a shoulder at 8984 eV and a pre-edge peak at 8976 eV. The pre-edge peak was related to 1s–3d electron transition occurring in the case of partially occupied 3d orbitals, which is possible for Cu^2+^ [71]. Taking at 8979 eV the K-edge energy of metallic copper (Cu^0^), we observed a chemical shift of 5.7 eV in the measured sample. The pre-edge feature and the value of the chemical shift of the absorption edge suggest that copper is in the divalent oxidation state in the Me-S-H sample. The Fourier transform exhibited one main peak centered at c.a. 1.9 Å (not corrected for phase shift), and for longer distances, the signal faded out rapidly (Figure 4b,c). This is consistent with an average local environment for Cu atoms which is structurally ordered in the first coordination shell and is highly disordered at longer distances. A satisfactory fit of the EXAFS signal was obtained considering a backscattering contribution of a first-shell of four oxygen atoms disposed in square planar geometry around Cu atoms. The Cu-O distance was refined to be 1.945 ± 0.005 Å. Table 1 reports the k-range and R-range used in the fitting and the derived structural parameters. Results from data fitting cannot rule out the occurrence of two axial O atoms at longer distances (at 2.02 ± 0.01 Å and 2.33 ± 0.01 Å), compatible with a Jahn−Teller distorted-octahedral model typical of copper (II) complexes [72,73], that show a high atomic displacement factor, indicating a disordered configuration for the outermost coordination shell. These findings suggest that Cu coordination to oxygen atoms likely occurs on the Me-S-H surface and exclude Cu incorporation into more ordered crystallographic positions (also because of the significantly different ionic radii) as in the CaO layer or replacing Si in the *dreierketten* chain unit. 

**Figure 5:**
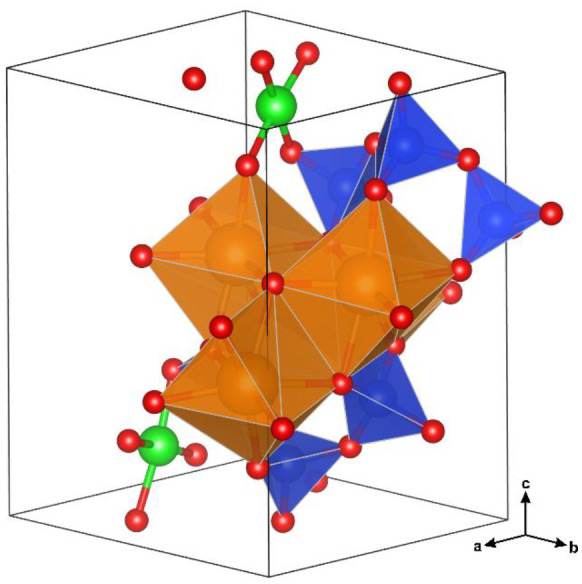
Unit cell content of the Cu-doped Me-S-H single-layer atomistic model showing the position of Ca (orange), Si (blue), O (red) and Cu (green) atoms. The population of single-layer atomistic models of nanocrystals was built from this unit cell along the *a* and *b* growth directions.

Further exploring the feasibility of Cu incorporation, a tentative modification of the single-layer atomistic model, previously discussed, was carried out by considering (i) the results obtained from EXASF analysis, thus introducing Cu atoms in the square planar geometry coordinated to four O atoms at the previously refined distance (as for the purpose of WAXTS pattern simulation, the introduction of the two highly disordered axial O atomic sites of the Cu octahedral configuration would have produced a negligible contribution to the calculated pattern, so that the four O atoms planar configuration was selected as an acceptable approximation to be introduced in the model.Nevertheless, the selected location for Cu atoms in the model may also allow the coordination with six O atoms typical of the Jahn−Teller distorted-octahedron); (ii) the stereochemical configuration of the model to identify a conceivable location for the Cu ion at the surface, possibly sharing two O atoms with the single-layer structure. The site of choice was selected (Figure 5) so that two of the four O atoms coordinated to Cu ions are the bridging O between two silicate paired tetrahedra and one apical O of Ca polyhedra (where a water molecule was located).

**Figure 6:**
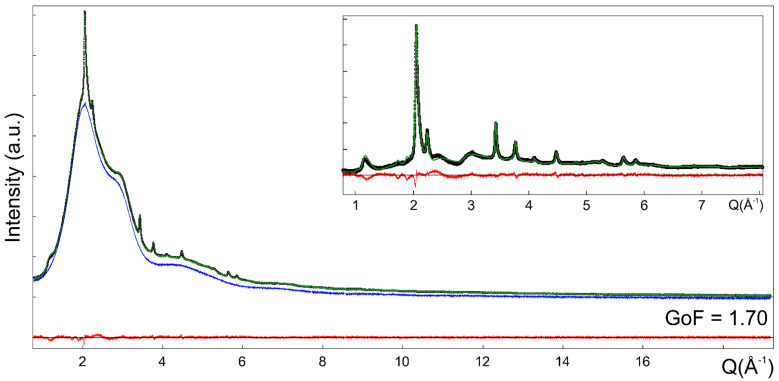
Synchrotron WAXTS data (black dots) of Me-S-H nanoparticles. The solid green line is the best fit obtained through the DSE using the Cu-doped single layer atomistic model; the red line is the residual between experimental and calculated patterns. The WAXTS pattern of the dispersing solution (blue trace) is added as a model component and scaled to the experimental data. The inset reports the WAXTS pattern and the DSE fit in a limited *Q* range by subtracting the scattering contribution of the solution.

Two O atoms were added and bound to Cu atoms, whereas two water molecules, too close to the added atoms, were removed (see the newly built unit cell in the Supplementary Materials). The single-layer atomistic model with Cu incorporation was then used to simulate the WAXTS pattern through the DSE. The model parameters (size and size dispersion, s.o.f. and Debye-Waller factors) were set equal to those previously refined in the SAXS/WAXTS-DSE analysis, whereas the Cu (and coordinated O) s.o.f. was set equal to the nominal value provided by the synthesis (Cu/Ca molar ratio = 0.1). At first, all model parameters were independently refined, providing a good fit of the experimental data. Slight variations in the Si s.o.f.s were observed, with those of particularly stable Si_p_ sites being close to s.o.f. = 0.86. Therefore, the refined occupancy of Si_p_ sites was fixed at 0.86, and that of Si_b_ sites was optimized by matching the calculated *Q*^n’^ with ^29^Si MAS-NMR results (as discussed in Section 3.4) and then fixed at 0.65 (with the numerical factor *A* = 0.44). Further refinement of the remaining model parameters led to the best fit (GoF = 1.70, Figure 6, Supplementary Table S1), showing a slight improvement with respect to the previous Cu-free model (refined site occupancy and Debye-Waller factors for each atomic species are reported in Table S3). The partial occupancy of Cu sites accounts for the limited and randomly distributed occurrence of Cu ions in the Me-S-H nanoparticles, for which the specific position suggested here may be a simplification of a more disordered configuration, as the quite high thermal parameter may indicate. The refined model parameters provided Cu/Ca and (Cu+Ca)/Si molar ratios of 0.07 and 0.91, respectively, close to the nominal ones (0.1 and 1, respectively) from the synthesis procedure.

Slight discrepancies between the modeled and nominal molar ratios can reasonably be ascribed to the ionic species occurring in the solution (i.e., extra Ca^2+^, Na^+^, NO_3_^−^) that may be weakly (randomly) bound to the nanoparticles, for which the atomistic model cannot account for. Despite the tentative construction of a Cu-doped C-S-H model, the excellent agreement between the calculated and experimental WAXTS patterns, combining XAS data in the atomistic model, shows the feasibility of structural incorporation of Cu ions into a C-S-H-based material.

## 4. Conclusions

Here we propose a comprehensive characterization of Cu-doped C-S-H nanoparticles in colloidal suspension by analyzing X-ray scattering data in the small- and wide-angle region through a unified modeling approach based on atomistic models and integrating structural details on the local chemical environment of selected atomic species. Results from different techniques converged into a consistent model providing detailed information from the atomic- to the nanoscale. Me-S-H nanoparticles result to be constituted by ultra-thin layers of a few tens of nanometers in diameter, with a structure resembling that of a single tobermorite layer made of 7-fold coordinated Ca atoms and decorated by defective silicate chains. Integrating the results from ^29^Si MAS-NMR and Cu K-edge XAS into the atomistic model, more detailed insights into the structural defectiveness were achieved. Notably, results show: (i) the occurrence of Si vacancies other than the sole bridging sites—pointing towards the lack of silicate chain fragments; (ii) effective Cu structural incorporation, thereby proving the feasibility of transition metals doping of calcium silicate hydrates, potentially opening new perspectives on materials engineering. The novel single-layer model enables to describe the size, morphology and structure of highly dispersed C-S-H-based nanoparticles showing the lack of any structural order between layers. This can be considered a limit case for which PCE polymers play a fundamental role to enhance the nanoparticles dispersion and stabilizing the colloidal suspension; nevertheless, this model can be readily applied to other C-S-H phases for which single tobermorite-like layers progressively stack up upon maturation and for which the high degree of stacking disorder is currently described by turbostratic models [29,54,56]. Further development of this model by introducing other structural defectiveness and Al incorporation is a promising approach to characterize nano-sized and defective Al-rich calcium silicate hydrates (C-A-S-H) occurring in blended cements and alkali activated materials. This work shows the potential of the combined SAXS/WAXTS-DSE approach, together with spectroscopic techniques, to analyze nanostructured cementitious materials, for which accessing the structural details at the atomic- and nanoscale is crucial to effectively manipulate the functional properties of relevant construction materials at the macro-scale.

## Figures and Tables

**Table 1:** Structural parameters obtained from Cu K-edge data analysis. Numbers in parenthesis: estimated uncertainties. N: number of atoms; S_0_^2^ passive electron reduction factor; R: interatomic distance; σ^2^: Debye-Waller factor; ΔE: energy shift; R-factor: agreement index.

Shell	N	S_0_^2^	R(Å)	σ^2^(Å^2^)	ΔE (eV)	R-Factor
Cu-O	4	0.90 (4)	1.945 (5)	0.0039(6)	5.9 (6)	0.002
k-range: 3.2–13.0; R-range: 1.1–2.3; k-weight: 1,2						

## Data Availability

All data is contained within the article.

## Supplementary Materials

The following are available online at https://www.mdpi.com/article/10.3390/nano12030342/s1. Preliminary analysis of the Me-S-H WAXTS pattern; Single-layer model unit cell optimization—computational details; Details on the simulation of Qn species from the atomistic model to describe the silicate chain connectivity; Unit cell parameters and fractional atomic coordinates used to build the atomistic models of C-S-H nanocrystals with Cu incorporation (Me-S-H model); Figure S1: Me-S-H/PCE WAXTS data; Figure S2: Preliminary DSE analysis of WAXTS data; Figure S3: Normalized XANES spectra of the Me-S-H/PCE sample measured as colloidal suspension and dried powder; Table S1: model parameters used in SAXS and WAXTS analyses; Table S2: Refined site occupancy (s.o.f.) and Debye-Waller B factors for each atomic species obtained from the best fit of WAXTS data of Me-S-H nanoparticles using the single-layer atomistic model; Table S3: Refined site occupancy (s.o.f.) and Debye-Waller B factors for each atomic species obtained from the best fit of WAXTS data of Me-S-H nanoparticles using the Cu-doped single-layer atomistic model.
