# Supplementary material for: An Atomistic Model Describing the Structure and Morphology of Cu-Doped C-S-H Hardening Accelerator Nanoparticles
Source: Nanomaterials (Basel). 2022 Jan 21;12(3):342. doi: 10.3390/nano12030342 (PMC8839642; doi:10.3390/nano12030342)
Supplement: Supplementary file 1 [file nanomaterials-12-00342-s001.zip › nanomaterials-1547960-supplementary.pdf]

nanomaterials

Article

An Atomistic Model Describing the Structure and Morphology of Cu-Doped C-S-H Hardening Accelerator Nanoparticles

Gregorio Dal Sasso [1,*], Maria Chiara Dalconi [2], Giorgio Ferrari [3], Jan Skov Pedersen [4], Sergio Tamburini [5], Federica Bertolotti [6], Antonietta Guagliardi [7], Marco Bruno [8], Luca Valentini [2] and Gilberto Artioli [2]

1 Institute of Geosciences and Earth Resources, National Research Council of Italy, Via G. Gradenigo 6, 35131, Padova, Italy
2 Department of Geosciences and CIRCe Centre, University of Padova, Via G. Gradenigo 6, 35131, Padova, Italy; mariachiara.dalconi@unipd.it (M.C.D.); luca.valentini@unipd.it (L.V.); gilberto.artioli@unipd.it (G.A.)
3 MAPEI Spa, R&D Laboratory, 20158, Milano, Italy; g.ferrari@mapei.it
4 Department of Chemistry and Interdisciplinary Nanoscience Center (iNANO), Aarhus University, Gustav Wieds Vej 14, 8000 Aarhus, Denmark; jsp@chem.au.dk
5 Institute of Condensed Matter Chemistry and Technologies for Energy, National Research Council of Italy, Corso Stati Uniti 4, 35127 Padova, Italy; sergio.tamburini@cnr.it
6 Department of Science and High Technology and To.Sca.Lab, University of Insubria, Via Valleggio 11, 22100 Como, Italy; federica.bertolotti@uninsubria.it
7 Institute of Crystallography and To.Sca.Lab, National Research Council of Italy, Via Valleggio 11, 22100 Como, Italy; antonella.guagliardi@ic.cnr.it
8 Department of Earth Sciences, University of Turin, Via Valperga Caluso 35, 10125, Torino, Italy; marco.bruno@unito.it

1. Preliminary Analysis of the Me-S-H WAXTS Pattern

The Me-S-H/PCE WAXTS pattern (violet trace in Figure S1) shows few and broad peaks compatible with those produced by a nanocrystalline tobermorite structure, as reported in previously developed models for C-S-H [1–7]. Notable in this case is the lack of the basal (002) reflection characteristic of the layer-to-layer distance periodicity of tobermorite structures in the low-angle region of the WAXTS pattern (0.45–0.50 Å-1 Q range).

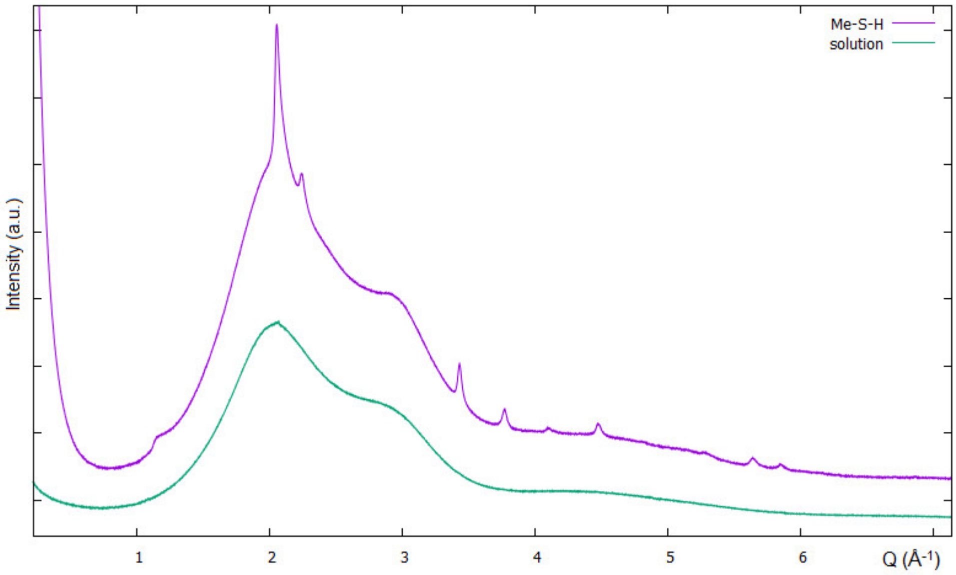

Figure S1. WAXTS data of Me-S-H sample (violet trace) and the dispersing solution (green trace) displayed in the Q range 0.22–7 Å^{-1}.

In the preliminary WAXTS/DSE analyses, atomistic models were built from previously reported tobermorite structures to simulate the WAXTS pattern of Me-S-H through

the Debye scattering equation. The unit cell of both tobermorite 11 Å from Merlino et al. (2001) [8] and tobermorite 14 Å from Bonaccorsi et al. (2005) [9] were used as building block to generate a population of atomistic models of disc-shaped nanocrystals at increasing size in the a-b plane and maintaining the dimension of a single unit cell along the c direction, to account for the nanoparticles size and morphology obtained from SAXS analysis (details in section 3.2 in the main text). This enables to generate tobermorite-like double-sheet nanocrystal models with a layer-to-layer distance of ~11 Å and ~14 Å, respectively. The a and b cell parameters for both structures were optimized against the WAXTS data with respect to those of the published structure. The new cell parameters for Tobermorite 11 Å are: a = 6.7118 Å, b = 7.3397 Å, c = 22.6800 Å, α = 90.00 °, β = 90.00 °, γ = 123.18 °. The new cell parameters for Tobermorite 14 Å are: a = 6.6612 Å, b = 7.3436 Å, c = 27.9870 Å, α = 90.00 °, β = 90.00 °, γ = 123.25 °. A preliminary single layer model was built using half (along the c axis) of the unit cell content of the tobermorite 11 Å [8] with adjusted a and b cell parameters (as previously reported for tobermorite 11 Å model), resulting in a single Ca layer sandwiched between silicate chains and water molecules. Preliminary DSE simulations using the tobermorite 11 Å (Figure S2a), 14 Å (Figure S2b) and the single-layer (Figure S2c) atomistic models were computed from a population of nanocrystal models at equal number-based average diameter and diameter distribution, according to a lognormal distribution function (Diameter D = 11.17 nm, relative size dispersion σD/D = 0.57), full site occupancy and equal Debye Waller factors B = 1.25 Å2. The nanocrystals thickness is determined by the c dimension of the single unit cell, resulting in a double layer structure in the case of Tobermorite 11 Å and 14 Å, and a single layer in the third case, with the nanoparticles thickness of 2.27 nm, 2.80 nm and 1.13 nm, respectively. The WAXTS pattern of the dispersing solution was added as a model component and scaled to the experimental data.

The goodness of fit (GoF) obtained from the DSE simulation, at equal model parameters, using the Tobermorite 11 Å, 14 Å, and the single layer models is 6.89, 7.22 and 3.34 showing a significant improvement (GoF closer to 1) of the agreement between the experimental and computed patterns using the single layer atomistic model. This is also clearly visible in the residual profiles (red traces in figure S2).

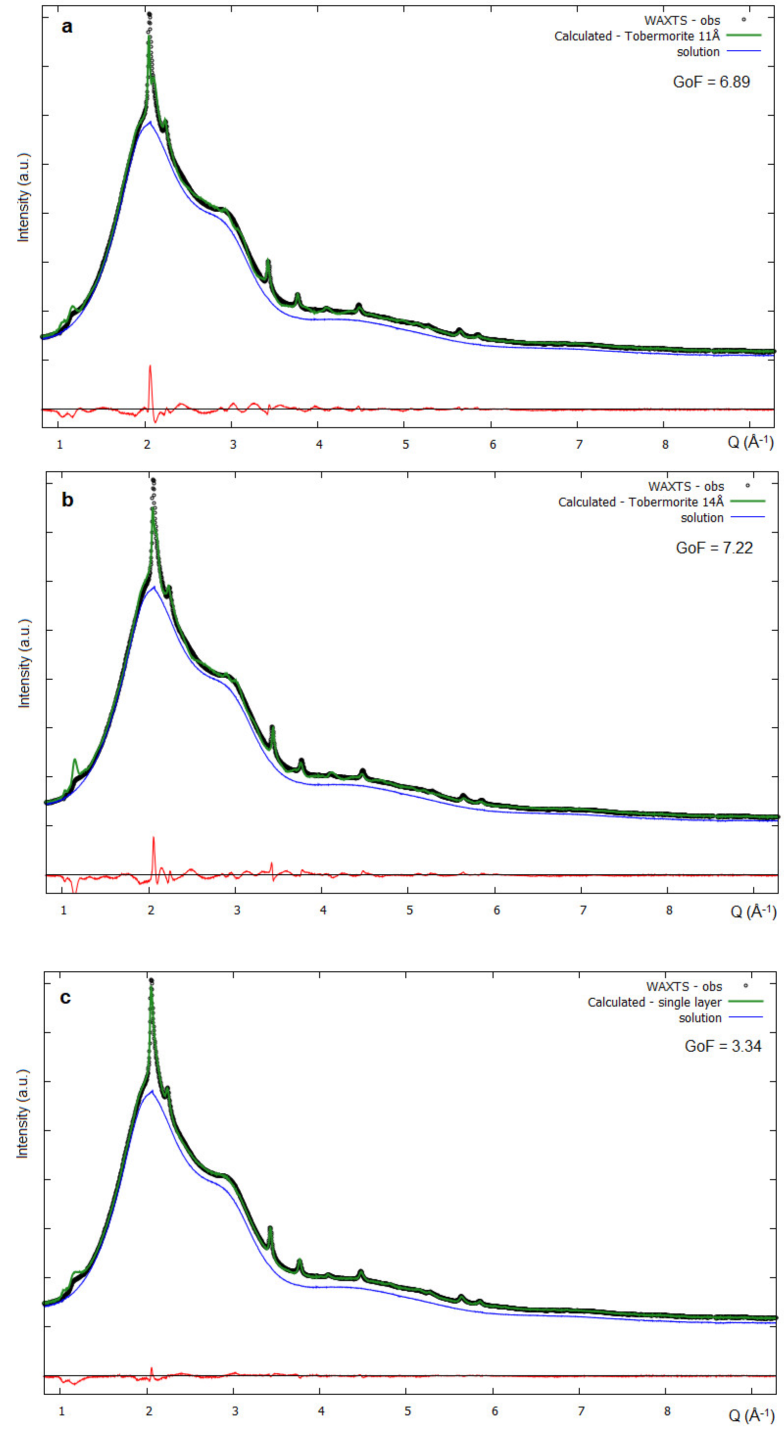

Figure S2. WAXTS data (black dots, data displayed in the Q range 0.8–9 Å^{-1}) of Me-S-H sample and DSE analysis using (**a**) the Scheme 11. Å, (**b**) the structural model of Tobermorite 14 Å, (**c**) the single-layer atomistic model. The WAXTS pattern of the dispersing solution (blue trace) is added as a model component and scaled to the experimental data. The red line shows the residual between experimental and calculated patterns. The goodness of fit (GoF) is reported for each simulation.

2. Single-Layer Model Unit Cell Optimization - Computational Details

The *ab initio* CRYSTAL14 code [10,11] was employed, which implements the Hartree-Fock and Kohn-Sham self-consistent field (SCF) method for the study of periodic systems [12]. The simulations were performed using the 2D periodic slab model, consisting of a film formed by a set of atomic layers parallel to the *hkl* crystalline plane of interest [13]. All the calculations were performed at the DFT (Density Functional Theory) level. In the

Density Functional approach, the B3LYP Hamiltonian was adopted [14–16], which contains a hybrid Hartree-Fock/Density-Functional exchange term and already shown to provide accurate results for structural and dynamical properties of hydroxyapatite.

In CRYSTAL, the multi-electronic wave-function is constructed as an anti-symmetrized product (Slater determinant) of mono-electronic crystalline orbitals (COs) which are linear combinations of local functions (i.e.: atomic orbitals, AOs) centered on each atom of the crystal. In turn, AOs are linear combinations of Gaussian-type functions (GTF, the product of a Gaussian times a real solid spherical harmonic to give *s*-, *p*- and *d*-type AOs). In this study, silicon, oxygen and hydrogen were described by (8*s*)-(6311*sp*)-(1*d*), (8*s*)-(411*sp*)-(1*d*) and (31*s*)-(1*p*) contractions, respectively. For calcium atoms, the first 10 electrons were described with the Hay−Wadt small corepseudo-potential, [HAYWSC]-(31*sp*). A double split valence basis set of GTFs was employed for describing the remaining 10 electrons, with 0.500 Bohr^{-2} as the exponent of the most diffuse *sp* shell [17]. The thresholds controlling the accuracy in the evaluation of Coulomb and exchange integrals (ITOL1, ITOL2, ITOL3, ITOL4 and ITOL5, see Dovesi *et al.* [11]) were set to 10^{-7} (ITOL1 to ITOL4) and 10^{-16} (ITOL5). The threshold on the SCF energy was set to 10^{-7} Hartree. In the adopted package the DFT exchange and correlation contributions are evaluated by numerically integrating functions of the electron density and of its gradient over the cell volume. The choice of the integration grid is based on an atomic partition method, originally developed by Becke [18]. In the present study, a *pruned* (75, 974) *p* grid was adopted (XLGRID in the code [11]), which ensured a satisfactory accuracy in the numerically integrated electron charge density. The reciprocal space was sampled according to a Monkhorst-Pack mesh [19] with shrinking factor 8, corresponding to 34 **k** points in the first irreducible Brillouin zone.

Structure was optimized by using the analytical energy gradients with respect to atomic coordinates and lattice parameters within a quasi-Newton scheme, combined with the Broyden-Fletcher-Goldfarb-Shanno scheme for Hessian updating [20–22]. Convergence was checked on energy, gradient components and nuclear displacements. The threshold on energy between two subsequent optimization steps was set to 10^{-7} Hartree; the thresholds on the root-mean-square of the gradient components and of the nuclear displacements were set to $3.0 \times \cdot 10^{-4}$ Hartree Bohr^{-1}; those on the maximum components of the gradients and displacements were set to $4.5 \times \cdot 10^{-4}$ Hartree Bohr^{-1}. The optimized fractional atomic coordinates are reported in the following unit cell; being the unit cell content fully explicit, the original space group of Tobermorite is replaced by the P1 sg. This unit cell is the building block used to generate the atomistic models of nanocrystals at increasing size, with growth directions along the a and b axis. To speed up the calculations, water molecules (W1-2-3-4) and OH^- ions were approximated to the O atoms.

Space Group: P1; a = 6.71184 Å; b = 7.33967 Å; c = 12.3558 Å; α = 90.0 °; β = 90.0 °; γ = 123.18 °

Atom	relative *x*	relative *y*	relative *z*
Ca	0.51510000	0.43280000	0.58093899
Ca	0.49990000	0.92280000	0.57921101
Ca	0.99990000	0.92280000	0.42094699
Ca	0.01510000	0.43280000	0.41921801
Si	0.00890100	0.39229200	0.66100900
Si	0.01148099	0.97462999	0.66751700
Si	0.51147299	0.97461101	0.33266202
Si	0.50892001	0.39228798	0.33913702
Si	0.65771399	0.75732701	0.17632902
Si	0.15771298	0.75732701	0.82382902
O	0.43364401	0.89867999	0.77836901
O	0.01573300	0.51437801	0.77482400
O	0.01532001	0.86902801	0.78837701
O	0.01272801	0.18635201	0.71140900

O	0.77095301	0.30623099	0.59258099
O	0.77305599	0.81455699	0.59989900
O	0.24043702	0.54503199	0.58786801
O	0.24023702	0.04342801	0.59485800
O	0.74022500	0.04338400	0.40531700
O	0.74043601	0.54505100	0.41228300
O	0.27306001	0.81458899	0.40029699
O	0.27095402	0.30622999	0.40754501
O	0.51278400	0.18635402	0.28874900
O	0.51525699	0.86895000	0.21181001
O	0.51580201	0.51435301	0.22530201
O	0.93364799	0.89876600	0.22178400
O	0.64855000	0.73087299	0.04335900
O	0.14853999	0.73083899	0.95679501
O (W1)	0.53933099	0.48088798	0.77571600
O (W1)	0.03935800	0.48091001	0.22446001
O (W2)	0.13071499	0.38201499	0.01932902
O (W3)	0.71382098	0.38129699	0.04961901
O (W4)	0.23881399	0.07497701	0.05062098
O (W2)	0.63080100	0.38199501	0.98081801
O (W3)	0.21397199	0.38140499	0.95051202
O (W4)	0.73894401	0.07497801	0.94945699
H	0.18141700	0.61915100	0.97496202
H	0.68135100	0.61913702	0.02518798
H	0.57653501	0.40891298	0.83142999
H	0.66977400	0.63312801	0.78305100
H	0.07643501	0.40886600	0.16873300
H	0.16986298	0.63312098	0.21709598
H	0.56233300	0.96996399	0.83259900
H	0.06230699	0.96995599	0.16753999
H	0.65188702	0.25936001	0.98019900
H	0.79173199	0.51188501	0.98625499
H	0.34112400	0.36910301	0.98102199
H	0.24818601	0.39521901	0.87270499
H	0.69920900	0.95933199	0.00000000
H	0.91530599	0.16717199	0.94420100
H	0.15183400	0.25939501	0.01996298
H	0.29163400	0.51192400	0.01390402
H	0.84108702	0.36907801	0.01917902
H	0.74779501	0.39501600	0.12744598
H	0.19887902	0.95939900	0.00000000
H	0.41519100	0.16709900	0.05580599

3. Details on the Simulation Of Q^n Species from the Atomistic Model to Describe the Silicate Chain Connectivity

Knowing the structure of the entire population of atomistic models, the Q^n relative abundance (assuming the full occupancy of Si sites) was easily calculated, as each silicate chain is formed by two Q^1 sites (the first and the last tetrahedron), a number of Q^{2b} sites equal to the number of Si_b atoms and a number of Q^{2p} sites equal to the number Si_p atoms minus the Q^1 sites. More precisely, considering that each unit cell contains two Si_b and four Si_p atoms, knowing the number of unit cells used to build each atomistic model and the number of (complete) silicate chains (determined by the shape of the models), the number of Q^n sites for each nanocrystal model is:

$$Q^1 = 2Nch$$

$$Q^{2b} = 2Nuc$$

$$Q^{2p} = 4Nuc - 2Nch$$

where *Nch* is the number of silicate chains and *Nuc* the number of unit cells for each atomistic nanocrystal model. When introducing a vacancy in one of the Si atomic site the neighbouring Q^2 sites became Q^1 sites. Since the point vacancy at certain Si sites is accounted for, on average, by the refined occupancy factor (0 < s.o.f. < 1, site vacancy = 1 - s.o.f.), new $Q^{n\prime}$ were calculated from Q^n by considering the refined vacancies at Si sites. The arrangement of Si vacancies was constrained (reducing the degree of freedom of the system) by the assumption that the conversion of a Q^{2b} site into Q^1 is highly improbable, implying the vacancy of a Si_p atom maintaining a dandling Si_b. This is supported by previous studies on C-S-H; indeed, even though vacancies at silicate bridging sites are proved to be energetically favoured with respect to the paired ones [2], the occurrence of Si_p vacancy together with the partial replacement of silicate bridging and paired sites with calcium and hydroxyl groups, respectively, is emerging in the recent literature, with a number of different configurations at similar energies that highlights the disorder nature of the structure with randomly distributed local defects [23,24]. With such constrain, $Q^{2b\prime}$ abundance is easily calculated as directly related to the occupancy of Si_b atomic site, which is separately refined. On the other hand, decoupling the contribution of Q^1 and Q^{2p} sites is less straightforward, as their relative abundance, accounted for by the Si_p atomic site, varies at increasing both the Si_b and Si_p vacancies. Considering these aspects, $Q^{n\prime}$ were calculated as follow:

$$Q^{1\prime} = Q^1 \mathrm{Si}_{p_{Occ}} + 2\, Q^{2b}(1 - \mathrm{Si}_{b\_Occ})A$$

$$Q^{2b\prime} = Q^{2b}(\mathrm{Si}_{b\_Occ})$$

$$Q^{2p\prime} = Q^{2p} \mathrm{Si}_{p_{Occ}} - 2\, Q^{2b}(1 - \mathrm{Si}_{b\_Occ})A$$

where Q^1, Q^{2b} and Q^{2p} are the Q^n sites previously defined at full Si sites occupancy, Si_{p_Occ} and Si_{b_Occ} are the refined Si_p and Si_b site occupancy factors (Si_{p_Occ} = 0.84 Si_{b_Occ} = 0.64 from WAXTS-DSE analysis), respectively. The numerical factor *A*, ranging from 0 to 1, modulates the number of newly generated Q^1 sites taking into account adjoining Si vacancies: if *A* =1 for each vacancy at Si_b site, two Q^1 will form at the expense of Q^{2p} sites meaning that vacancies are not contiguous. If neighbouring vacancies occurs, so that longer fragments of the silicate chain are missing, each Si bridging site vacancy will generate less than two (on average) Q^1 sites, implying $A < 1$; this includes also Si_b vacancy at the edge of the nanocrystals near Q^1 sites. The abundance of $Q^{2p\prime}$ sites results from the previously calculated Q^{2p} multiplied for its occupancy minus the newly generated Q^1 sites. The total relative abundance of $Q^{n\prime}$ sites was calculated as the sum of $Q^{n\prime}$ for each nanocrystal atomistic model weighted for its number-based size distribution fraction. The numerical factor *A* was optimized by minimizing the residual sum of squares (RSS) calculated between the total relative abundance of $Q^{n\prime}$ and the experimental one.

4. Unit cell parameters and fractional atomic coordinates used to build the Atomistic Models of C-S-H Nanocrystals with Cu Incorporation (Me-S-H Model)

Space Group: P1; a = 6.71184 Å; b = 7.33967 Å; c = 12.3558 Å; α = 90.0 °; β = 90.0 °; γ = 123.18 °

Atom	relative *x*	relative *y*	relative *z*
Cu	0.67427840	0.27583540	0.15225900
Cu	0.17355713	0.27635201	0.84700900
Ca	0.51510000	0.43280000	0.58093899
Ca	0.49990000	0.92280000	0.57921101

Ca	0.99990000	0.92280000	0.42094699
Ca	0.01510000	0.43280000	0.41921801
Si	0.00890100	0.39229200	0.66100900
Si	0.01148099	0.97462999	0.66751700
Si	0.51147299	0.97461101	0.33266202
Si	0.50892001	0.39228798	0.33913702
Si	0.65771399	0.75732701	0.17632902
Si	0.15771298	0.75732701	0.82382902
O	−0.15357280	0.09635201	0.89830201
O	0.34733099	0.38388798	0.98260900
O	0.37278400	0.14135402	0.07522223
O	0.83682098	0.34029699	0.01541901
O	0.43364401	0.89867999	0.77836901
O	0.01573300	0.51437801	0.77482400
O	0.01532001	0.86902801	0.78837701
O	0.01272801	0.18635201	0.71140900
O	0.51278400	0.18635402	0.28874900
O	0.51525699	0.86895000	0.21181001
O	0.51580201	0.51435301	0.22530201
O	0.93364799	0.89876600	0.22178400
O	0.77095301	0.30623099	0.59258099
O	0.77305599	0.81455699	0.59989900
O	0.24043702	0.54503199	0.58786801
O	0.24023702	0.04342801	0.59485800
O	0.74022500	0.04338400	0.40531700
O	0.74043601	0.54505100	0.41228300
O	0.27306001	0.81458899	0.40029699
O	0.27095402	0.30622999	0.40754501
O	0.64855000	0.73087299	0.04335900
O	0.14853999	0.73083899	0.95679501
O (W1)	0.47733099	0.44388798	0.77571600
O (W1)	−0.02293580	0.44091001	0.22446001
O (W2)	0.13071499	0.38201499	0.01932902
O (W2)	0.63080100	0.38199501	0.98081801

Table 1. SAXS analysis based on polydisperse flat cylindrical discs: number-based average disc diameter (D), its relative dispersion (σD/D), number-based average thickness (T) and its relative dispersion (σT/T) according to a Schulz distribution. DSE-based analysis of SAXS and WAXTS data based on atomistic models (single layer model referring to sections 3.2.2, 3.3 and figure 2, Cu-doped single layer model referring to section 3.5 and figure 6): number-based average disc diameter (D), its relative dispersion (σD/D), number-based average thickness (T) and its relative dispersion (σT/T) according to a lognormal distribution. * indicates fixed parameters during the refinement; the nanoparticles thickness in the DSE modelling is not refinable as corresponding to the single layer thickness.

**Conventional SAXS modelling**		**D (nm)**	**σD/D**	**T (nm)**	**σT/T**	**χ^2**	**size distribution function**		
SAXS		9.78	0.49	1.17	0.05*	7.68	Schulz		
Debye Scattering Equation modelling	Atomistic model	D (nm)	σD/D	T (nm)	σT/T	GoF	size distribution function	s.o.f.	B factor (Å^2)
SAXS-DSE	Single layer	11.24	0.59	1.13*	-	1.13	Lognormal	1.00*	1.25*
Porod/WAXTS-DSE	Single layer	11.24*	0.59*	1.13*	-	3.11	Lognormal	1.00*	1.25*
WAXTS-DSE	Single layer	11.24*	0.59*	1.13*	-	1.74	Lognormal	As in Table S2	As in Table S2

WAXTS-DSE	Cu-doped single layer	11.24*	0.59*	1.13*	-	1.70	Lognormal	As in Table S3	As in Table S3

Table 2. Refined site occupancy (s.o.f.) and Debye-Waller B factors for each atomic species obtained from the best fit of WAXTS data of Me-S-H nanoparticles using the single-layer atomistic model. Si_b and Si_p were treated as separate atomic species as well as O atoms in the silicate chains [O(Si)], in the Ca layer [O(Ca)] and those approximating the OH^- ions at the apical O of silicate bridging tetrahedra and water molecules (W1, W2, W3 and W4). All model parameters were separately refined; s.o.f. of O (Si) and O (H) were fixed as equal to those of Si_p and Si_b, respectively.

**Atom**	**S.o.f.**	**B factor (Å^2)**
Ca	1.000	1.257
Si_p	0.840	1.202
Si_b	0.638	2.887
O (Si)	0.840	1.202
O (Ca)	1.000	1.258
O (H)	0.638	3.869
O (W1)	0.999	2.828
O (W2)	0.536	9.880
O (W3)	0.779	7.230
O (W4)	0.010	5.416

Table 3. Refined site occupancy (s.o.f.) and Debye-Waller B factors for each atomic species obtained from the best fit of WAXTS data of Me-S-H nanoparticles using the Cu-doped single-layer atomistic model. Si_b and Si_p were treated as separate atomic species as well as O atoms coordinated to Cu^{2+} ions [O(Cu)], those in the silicate chains [O(Si)], in the Ca layer [O(Ca)] and those approximating the OH^- ions at the apical O of silicate bridging tetrahedral and water molecules (W1, W2). All model parameters were separately refined with the exception of s.o.f. of Si_b atoms and the apical O atoms of the bridging silicate tetrahedra [O(H)], as determined through the simulation of $Q^{n'}$ species determined by ^{29}Si MAS-NMR. s.o.f. of O (Si) was fixed as equal to that of Si_p.

**Atom**	**S.o.f.**	**B factor (Å^2)**
Cu	0.143	4.500
Ca	1.000	1.257
Si_p	0.856	1.204
Si_b	0.653	4.272
O (Cu)	0.120	4.953
O (Si)	0.856	1.200
O (Ca)	1.000	1.258
O (H)	0.653	5.000
O (W1)	0.998	5.918
O (W2)	0.559	9.928

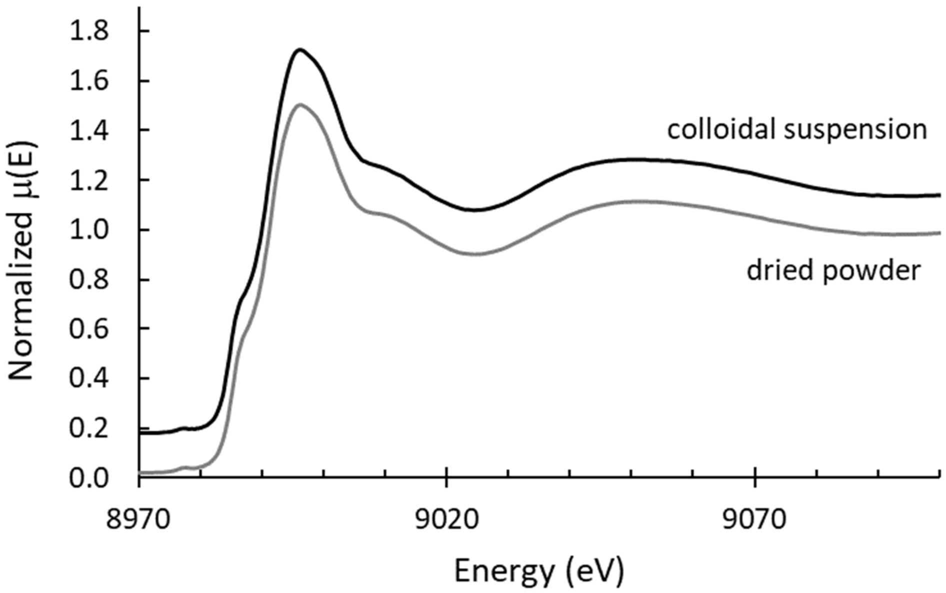

Figure S3. Normalized XANES spectra of the Me-S-H/PCE sample measured as colloidal suspension and dried powder. The colloidal suspension spectrum is shifted vertically for clarity.

References:

1. Gaboreau, S.; Grangeon, S.; Claret, F.; Ihiawakrim, D.; Ersen, O.; Montouillout, V.; Maubec, N.; Roosz, C.; Henocq, P.; Carteret, C. Hydration Properties and Interlayer Organization in Synthetic C-S-H. *Langmuir* **2020**, 36, 9449–9464.
2. Kumar, A.; Walder, B.J.; Kunhi Mohamed, A.; Hofstetter, A.; Srinivasan, B.; Rossini, A.J.; Scrivener, K.; Emsley, L.; Bowen, P. The Atomic-Level Structure of Cementitious Calcium Silicate Hydrate. *J. Phys. Chem. C.* **2017**, 121, 17188–17196.
3. Grangeon, S.; Claret, F.; Roosz, C.; Sato, T.; Gaboreau, S.; Linard, Y. Structure of nanocrystalline calcium silicate hydrates: insights from X-ray diffraction, synchrotron X-ray absorption and nuclear magnetic resonance. *J. Appl. Crystallogr*. **2016**, 49, 771–783.
4. Cuesta, A.; Zea-Garcia, J.D.; Londono-Zuluaga, D.; De La Torre, A.G.; Santacruz, I.; Vallcorba, O.; Dapiaggi, M.; Sanfélix, S.G.; Aranda, M.A.G. Multiscale understanding of tricalcium silicate hydration reactions. *Sci. Rep.* **2018**, 8, 1–11.
5. Cuesta, A.; Santacruz, I.; De la Torre, A.G.; Dapiaggi, M.; Zea-Garcia, J.D.; Aranda, M.A.G. Local structure and Ca/Si ratio in C-S-H gels from hydration of blends of tricalcium silicate and silica fume. *Cem. Concr. Res*. **2021**, 143, 106405.
6. Li, J.; Geng, G.; Myers, R.; Yu, Y.S.; Shapiro, D.; Carraro, C.; Maboudian, R.; Monteiro, P.J.M. The chemistry and structure of calcium (alumino) silicate hydrate: A study by XANES, ptychographic imaging, and wide- and small-angle scattering. *Cem. Concr. Res*. **2019**, 115, 367–378.
7. Renaudin, G.; Russias, J.; Leroux, F.; Frizon, F.; Cau-dit-Coumes, C. Structural characterization of C–S–H and C–A–S–H samples—Part I: Long-range order investigated by Rietveld analyses. J. *Solid State Chem*. **2009**, 182, 3312–3319.
8. Merlino, S.; Bonaccorsi, E.; Armbruster, T. The real structure of tobermorite 11A: normal and anomalous forms, OD character and polytypic modifications. *Eur. J. Mineral*. **2001**, 13, 577–590.
9. Bonaccorsi, E.; Merlino, S.; Kampf, A.R. The Crystal Structure of Tobermorite 14 A (Plombierite), a C-S-H Phase. *J. Am. Ceram. Soc*. **2005**, 88, 505–512.
10. Dovesi, R.; Orlando, R.; Civalleri, B.; Roetti, C.; Saunders, V.R.; Zicovich-Wilson, C.M. CRYSTAL: a computational tool for the ab initio study of the electronic properties of crystals. Zeitschrift für Krist. *Cryst. Mater*. **2005**, 220, 571–573.
11. Dovesi, R.; Saunders, V.R.; Roetti, C.; Orlando, R.; Zicovich-Wilson, C.M.; Pascale, F.; Civalleri, B.; Doll, K.; Harrison, N.M.; Bush, I.J.; et al. CRYSTAL14 User's Manual. Univ. Torino **2014**, 382.
12. Pisani, C.; Dovesi, R.; Roetti, C. *Hartree-Fock ab-initio treatment of crystalline systems, Lecture Notes in Chemistry*; Springer: Berlin, Heidelberg, New York, **1988.**
13. Dovesi, R.; Civalleri, B.; Roetti, C.; Saunders, V.R.; Orlando, R. Ab Initio Quantum Simulation in Solid State Chemistry. *Reviews in computational chemistry*; **2005**; pp. 1–125.
14. Becke, A.D. Density-functional thermochemistry. III. The role of exact exchange. *J. Chem. Phys*. **1993**, 98, 5648–5652.
15. Lee, C.; Yang, W.; Parr, R.G. Development of the Colle-Salvetti correlation-energy formula into a functional of the electron density. *Phys. Rev. B.* **1988**, 37, 785–789.

16. Stephens, P.J.; Devlin, F.J.; Chabalowski, C.F.; Frisch, M.J. Ab Initio Calculation of Vibrational Absorption and Circular Dichroism Spectra Using Density Functional Force Fields. *J. Phys. Chem*. **1994**, 98, 11623–11627.
17. Corno, M.; Busco, C.; Civalleri, B.; Ugliengo, P. Periodic ab initio study of structural and vibrational features of hexagonal hydroxyapatite Ca10(PO4)6(OH)2. *Phys. Chem. Chem. Phys*. **2006**, 8, 2464.
18. Becke, A.D. Density-functional exchange-energy approximation with correct asymptotic behavior. *Phys. Rev. A*. **1988**, 38, 3098–3100.
19. Monkhorst, H.J.; Pack, J.D. Special points for Brillouin-zone integrations. *Phys. Rev. B*. **1976**, 13, 5188–5192.
20. Civalleri, B.; D'Arco, P.; Orlando, R.; Saunders, V.R.; Dovesi, R. Hartree–Fock geometry optimisation of periodic systems with the Crystal code. Chem. *Phys. Lett*. **2001**, 348, 131–138.
21. Doll, K. Implementation of analytical Hartree–Fock gradients for periodic systems. Comput. *Phys. Commun*. **2001**, 137, 74–88.
22. Doll, K.; Saunders, V.R.; Harrison, N.M. Analytical Hartree-Fock gradients for periodic systems. *Int. J. Quantum Chem*. **2001**, 82, 1–13.
23. Chiang, Y.; Chang, S.-W. Bridging the gap between NMR measured mean silicate chain length and nano-scale silicate polymorphism of calcium silicate hydrates. *Cem. Concr. Res*. **2021**, 140, 106268.
24. Kunhi Mohamed, A.; Parker, S.C.; Bowen, P.; Galmarini, S. An atomistic building block description of C-S-H - Towards a realistic C-S-H model. *Cem. Concr. Res*. **2018**, 107, 221–235.
